# Nutritional status of people who inject drugs in Coastal Kenya: a cross-sectional study

**DOI:** 10.1186/s40795-024-00851-z

**Published:** 2024-04-04

**Authors:** Valentine Budambula, Moses Ngari, Nancy L.M. Budambula, Aabid A. Ahmed, Tom Were

**Affiliations:** 1 Department of Environment and Health Sciences, Technical University of Mombasa, Mombasa, P. O. Box 90420-80100, Kenya; 2 KEMRI-Wellcome Trust Research Programme, Kilifi, Kenya; 3 Department of Public Health, Pwani University, Kilifi, Kenya; 4 Department of Biological Sciences, University of Embu, Embu, Kenya; 5 Bomu Hospital, Mombasa, P.O Box 95683-80106, Kenya; 6 Department of Microbiology and Parasitology, Masinde Muliro University of Science and Technology, Kakamega, Kenya

**Keywords:** Under nutrition, Injection drug use, BMI, MUAC and anaemia

## Abstract

**Introduction:**

Despite documentation on injection drug use (IDU) in Kenya, the nutritional status of people who inject drugs (PWIDs) is under-explored. Elsewhere studies report under-nutrition among PWIDs which is attributed to food insecurity; competing priorities between drugs and food supply; chaotic lifestyle; reduced food intake; substance use induced malnutrition due to inflammation and comorbidities.

**Methods:**

This was a cross-sectional study that sought to assess the nutritional status of PWIDs in Coastal Kenya. We recruited 752 participants of whom 371(49%) were on IDUs and 75 non-IDUs and 306 non-drug users using respondent driven sampling, traditional snowball, makeshift outreach and purposive sampling methods.

**Results:**

More than one half of the participants (56%) had BMI classified as normal while 35% had BMI < 18.5. The proportion with BMI < 18.5 was higher among IDUs (46%) compared to the non-IDUs (33%) and non-drug users (23%) at *P* < 0.001. Using the mid upper arm circumference (MUAC), 17% were classified as underweight and the proportion was lowest (11%) among non- drugs users compared to 22% among IDUs (*P* < 0.001). However, the IDUs had lower proportion of overweight (8.1%) compared to 55% among the non- drug users. The proportion with low waist-for-hip ratio was highest among the IDUs (74%) while high waist-for-hip ratio was lowest in the same group of IDUs (11%) at *P* < 0.001. One half (50%), of the participants had no signs of anaemia, (47%) had mild/moderate anaemia while 21 (2.8%) had severe anaemia. However, IDUs were more likely to be overweight based on waist circumference as a parameter. The IDUs had the highest proportion (54%) of mild to moderate anaemia compared to non-IDUs (37%) and 40% non- drug users (*P* < 0.001). In the multivariable models, IDUs (aRRR 2.83 (95%CI 1.84‒4.35)) and non-IDUs (aRRR 1.42 (95%CI 1.07‒1.88)) compared to non- drug users were positively associated with BMI < 18.5. Being an IDU was positively associated with mild or moderate anaemia (aRRR 1.65 (95%CI 1.13‒2.41)) while non-IDUs were positively associated with severe anaemia (aRRR 1.69 (95%CI 1.16‒2.48)).

**Conclusion:**

A significant proportion of the participants were under-nourished with those injecting drugs bearing the heaviest brunt. Being an IDU was positively associated with the low BMI, MUAC, waist for hip ratio and mild or moderate anaemia but high waist circumference. People who inject drugs have high risk for under-nutrition and should be targeted with appropriate interventions.

**Supplementary Information:**

The online version contains supplementary material available at 10.1186/s40795-024-00851-z.

## Introduction

Substance abuse and addiction remains a major public health problem both globally and in Kenya [1, 2]. Under-nutrition among injecting drug users (IDUs) has been attributed to food insecurity; competing priorities between drugs and food supply; chaotic lifestyle; reduced food intake; substance use induced malnutrition due to inflammation; and comorbidities [3–7]. In Vancouver (Canada) and Chennai (India) for example, between 65% and 70% of drug users reported having experienced food insecurity as well as under-nutrition [4, 8]. Most people who use drugs (PWUDs) face competing demands between addiction and subsistence which in return hinders food selection. Consequently, their diets have been reported to consist of foods of low nutritive value such as fatty diets or ready to eat sweet and salty snacks. Fruits, vegetables, grains and dairy products are hardly included in their diets [9–13]. In South Africa, for instance low levels of vitamin D and calcium have been reported among adolescents with alcohol dependence [14].

Reduced food intake in this sub-population can be attributed to suppressed appetite, selective appetite and distorted food taste. Appetite suppression has been documented among khat users [15, 16] and people who use tobacco. Nicotine, the active ingredient in tobacco has been shown to enhance leptin level resulting in suppressed appetite [17]. Nicotine also reduces food intake as a result of activation of proopiomelanocortin (POMC) neurons which suppress appetite [18, 19] or impaired taste [20]. Heroin users in Australia have been reported to be underweight [21]. This is probably due to the fact that opiates influence thyroid function by elevating total triiodothyronine (T3) while lowering T3 Resin uptake (T3RU) and free thyroxine (T4) levels [22]. Equally, methamphetamine use negatively affects both total cholesterol levels and body mass index [23]. Reduced food intake can also be attributed to dental disorders and periodontal disease. The two conditions if left untreated affects food chewing and digestion [24].

Overall, substance use alters eating habits leading to poor dietary patterns such as poorly structured eating schedule or consuming fewer meals per week [8, 4]. Even though it is reported that 34.4% of residents of Mombasa County are using at least one substance of abuse [25], there is paucity of data on the nutritional status of drug users especially among the IDUs. In this study, we sought to assess the nutritional status of people who inject drugs also known as injection drug users in Coastal Kenya based on anthropometric measurements. In the context of this study, drug use has been operationalized to use of drugs for psychotropic rather than medical reasons.

## Methods

### Setting, design and study participants

This was a cross-sectional study carried out in 2012–2013. Part of the study findings have been presented in a thesis titled “Predictors of HIV and Pulmonary TB Infections among Injection Drug Users in Mombasa County, Kenya” [26]. Additionally portions of the study have been published [6, 27–30, 31]. We used respondent driven sampling, traditional snowball, makeshift outreach and purposive sampling methods to recruit the IDUs and the non-IDUS. The study recruited 752 participants of which 371(49%) were IDUs and 381 non-drug users.

The 371 IDUs were sampled from six constituencies of Mombasa County namely Kisauni, Mvita, Changamwe, Likoni, Jomvu and Nyali. These participants were enrolled through Bomu Hospital (in Changamwe) and its branches in Kisauni, Likoni and Jomvu. The 381 non-drug using participants were enrolled through Coast General Teaching & Referral Hospital located in Mvita and its outreach branches in Changamwe and Kisauni. Eligibility criteria were limited to adults residing in Mombasa County who were in the out-patient department and provided written informed consent. Additional criterion which applied to IDUs was having injected at least once a day in the previous one month as well as the presence of needle scars.

### Sample size determination

Sample size for the original study was estimated using the formula n= [(Z^2^) *p*(*q*)]/*d*^2^ [32]. Our *p* which is the proportion of drug abuse was estimated to be 0.5 based on the report that 54% of the residents of Mombasa County have ever used at least one substance of abuse in a lifetime [33]. The Z = 1.96 representing a two-tailed alpha of 0.05, q = 0.5 (1-0.5) and d the level of precision was 0.05. Based on this formula 384 IDUs and 384 non drug users were recruited. Among the 384 IDUs, 13 declined to take an HIV test and were not included in the final analysis. Among the 384 non- drug users three declined to take an HIV test and were excluded. The final analysis had 371 IDUs and 381 non-drug users. During data cleaning and analysis the later were further classified as non-IDUs (75) as they self-reported current use of non-injecting drugs and non-drug users (306).

### Data collection tools

Structured participant assisted questionnaire was used to document the socio-demographic characteristics of the respondents. Standing height (m) to the nearest cm and body weight (kg) to the nearest 100 g were measured using Health O meter. The BMI = [Weight (kg) ÷ Height (m) ^2^] was calculated. A tape measure was used to measure the bust, waist and hip circumference while the adult mid- upper arm circumference (MUAC) tape was used to assess MUAC. Hemoglobin levels and HIV tests we carried out in accordance with Ministry of Health guidelines [34, 35]. A biometric enrollment and verification kit was used at enrollment to avoid double admission into the study.

### Quantitative variables

Body mass index (BMI) was calculated as body weight divided by the square of height in metres and categorised into three groups following WHO guidelines [36]: underweight (BMI < 18.5), normal (BMI 18.5 to 24.9) and overweight/obese (BMI ≥ 25). Waist-for-hip ratio (WHR) was calculated as waist divided by hip measurement and categorised into three groups: low defined as WHR < 0.8 for female and < 0.95 for male; moderate as WHR 0.81 to 0.85 for female and 0.96 to 0.99 for male; and high as WHR ≥ 0.86 for female and ≥ 1.0 for male. The continuous MUAC in cm was categorised into three groups: Underweight was defined as MUAC < 23.9 cm; Normal as MUAC ≥ 24.0 to 28 cm for male and MUAC ≥ 24.0 to 28.3 cm for female; and Overweight/obese as MUAC ≥ 28.1 cm for male and MUAC ≥ 28.4 cm for female. The continuous hemoglobin in g/dl was used to calculate levels of anaemia in three groups: No anaemia was defined as hemoglobin ≥ 12 g/dl for female or ≥ 13 g/dl for male; mild/moderate anaemia as hemoglobin 8 to 11.9 g/dl for female and 8 to 12.9 g/dl for male and severe anaemia hemoglobin < 8.0 g/dl for both male & female.

### Statistical methods

All collected data were reviewed for outliers and data errors. Any data queries were resolved by checking the original data in the paper questionnaires. Cleaned data were exported to STATA statistical software for statistical analysis. Statistical analysis was performed using STATA (version 17.0; StataCorp, College Station, TX, USA) and R software (Version 4.2.0). The level of statistical significance was set at α < 0.05. All the variables included in the analysis were not missing any data.

Categorical variables were summarized as frequencies and proportions. Continuous variables were summarized as medians [interquartile range, IQR] because they were skewed. Proportions across the three groups (IDU users, non-IDUs and non-drug users) were compared using chi-square test. Medians for continuous variables were compared across the three groups using Kruskal-Wallis test and post hoc adjustment for each pairwise comparison conducted using the ‘holm’ method implemented by *FSA* package in R software [37].

To measure the effect of the level of drug use (IDU users, non-IDU users and non-drug users) on different anthropometric measurements, we conducted multinomial logistic regression and linear regression treating the recruiting hospital as cluster and used robust standard errors to control the effect of hospital-level clustering. Multinomial logistic regression analysis was conducted where the dependent variables were categorical with more than two levels; BMI group, waist-for-hip ratio, MUAC and anaemia levels. The measure of effect reported from the multinomial logistic regression models were log transformed regression coefficients into relative risk ratios (RRR). Height per meter was analyzed using linear regression because it had a normal distribution and there no recommended cutoffs. For both multinomial logistic and linear regression we conducted *base models* adjusting for age and sex only and multivariable models adjusting for a priori confounders (sex, age, education level, marital status, religion, monthly income, HIV status).

## Results

The study recruited 752 participants, 371(49%) were IDUs, 75 (10%) non-IDUs while 306 (41%) were non-drug users. Among the 371 on IDU drugs, 303 (82%) were injecting heroin, 61 (16%) cocaine while 7 (1.9%) were injecting both cocaine and heroin (Supplementary Table 1). The 371 participants had been injecting drugs for < 6 months (*N* = 48, 13%), 6 to 11 months (*N* = 51, 14%), 1 to 3 years (*N* = 138, 37%) and > 3 years (*N* = 134, 36%). The 75 non- IDUs self-reported current use of non-injecting drugs used like alcohol (*N* = 39/75, 52%), khat (*N* = 25, 33%), cigarettes (*N* = 25, 33%), rohypnol (*N* = 3, 4.0%), brown sugar (*N* = 3, 4.0%) and bhang (*N* = 5, 6.8%) as shown in Supplementary Table 2.

Their median (IQR) age was 32 [27–38] years and 435 (58%) were male. At least one third (*N* = 247, 33%) were currently married while 323 (43%) were Muslims. More than six in every ten participants (*N* = 479, 64%) had primary level or no education. Only one fifth (*N* = 159, 21%) were in formal employment while 213 (28%) earned > 240 US dollars per month. All the demographics stratified by drug use status are shown in Table 1. Overall, 297 (39%) of the participants were HIV infected out of which 43% were IDUs, 36% non-IDUs and 37% non-drug users (*P* = 0.23). Over two thirds of the HIV infected participants (*N* = 210/297, 68%) were on ARVs and the proportion of those taking ARVs was lower among IDUs (*P* = 0.007) as shown in Table 2.

**Table 1:** Socio-demographic Characteristic of IDUs, non IDUs and Non-DUs in in Coastal Kenya between 2012–2013

All participants		Drug Use Status			
Variables		Non- DUs	Non-IDUs	IDUs	*p*-value
	(*N* = 752)	(*N* = 306)	(*N* = 75)	(*N* = 371)	
Age; median [IQR] yrs	32 (27–38)	33 (27–40)	33 (27–41)	30 (27–35)	***0.02***
***Gender***, *n* (%)					
Male	435 (58)	114 (37)	50 (67)	271 (73)	***< 0.001***
Female	317 (42)	192 (63)	25 (33)	100 (27)	
***Education level***, *n* (%)					
None/Primary	479 (64)	163 (53)	46 (61)	270 (73)	**< 0.001**
Above primary	273 (36)	143 (47)	29 (39)	101 (27)	
***Marital status***, *n* (%)					
Never married	220 (29)	75 (25)	15 (20)	130 (35)	**< 0.001**
Currently married	247 (33)	139 (45)	34 (45)	74 (20)	
Divorced/separated/widowed	285 (38)	92 (30)	26 (35)	167 (45)	
***Religion***					
Catholic	208 (28)	93 (31)	29 (39)	86 (23)	**< 0.001**
Protestant	221 (29)	138 (45)	27 (36)	56 (15)	
Muslim	323 (43)	75 (25)	19 (25)	229 (62)	
***Income (US $/ month)***					
< 60	170 (23)	136 (44)	24 (32)	10 (2.7)	**< 0. 001**
60 to 120	160 (21)	93 (30)	32 (43)	35 (9.4)	
120 to 240	209 (28)	59 (19)	12 (16)	138 (37)	
> 240	213 (28)	18 (5.9)	7 (9.3)	188 (51)	
**Employment status**					
Self employed	491 (65)	144 (47)	43 (57)	304 (82)	**< 0.001**
Formally employed	159 (21)	72 (24)	23 (31)	64 (17)	
None/student	102 (14)	90 (29)	9 (12)	3 (0.8)	

The median age was compared using Kruskal–Wallis test all other *p*-values are from chi-square test, IQR; Interquartile range, the month income was collected in KES and converted into US dollar at exchange rate of one US dollar at KES 86.12 when study was conducted

**Table 2:** Clinical profile of IDUs, non-IDUs and non-DUs in Coastal Kenya between 2012–2013

All participants		Drug Use Status			
Variables		Non- DUs	Non-IDUs	IDUs	*p-value*
	(N = 752)	(N = 306)	(N = 75)	(N = 371)	
***HIV status***					
Negative	455 (61)	194 (63)	48 (64)	213 (57)	0.23
Positive	297 (39)	112 (37)	27 (36)	158 (43)	
***On ARVs (N = 297 HIV)***	201 (68)	88 (79)	17 (63)	96 (61)	0.007
***Duration of ARVs*** (N = 201)					
< 6 months	37 (18)	15 (17)	6 (35)	16 (17)	< 0.001
6 to 11 months	33 (16)	12 (13)	2 (11)	19 (20)	
12 to 35 months	68 (33)	14 (15)	2 (11)	52 (54)	
≥ 36 months	69 (33)	51 (55)	9 (47)	9 (9.4)	
**Clinical signs**					
Fever	309 (41)	80 (26)	23 (31)	206 (56)	< 0.001
Vomiting	230 (31)	52 (17)	9 (12)	169 (46)	< 0.001
Diarrhoea	212 (28)	52 (17)	13 (17)	147 (40)	< 0.001
Headache	273 (36)	76 (25)	18 (24)	179 (48)	< 0.001
Cough	346 (46)	109 (36)	36 (48)	201 (54)	< 0.001
***Chest examination***					
Normal/clear	585 (78)	258 (81)	59 (79)	278 (75)	< 0.001
Congested	99 (13)	46 (15)	12 (16)	41 (11)	
Wheezy	52 (6.9)	0	0	52 (14)	
Crepitation	16 (2.1)	12 (3.9)	4 (5.3)	0	

The medians are compared using Kruskal–Wallis test all other *p*-values are from chi-square test, IQR; Interquartile range

More than one half (*N* = 420, 56%) of the study participants had BMI classified as normal while 263 (35%) had BMI < 18.5. The proportion with BMI < 18.5 was higher among those on IDU (46%) compared to the non-IDUs (33%) and non-drug users (23%) at *P* < 0.001. Using the MUAC, 130 (17%) were classified as underweight and the proportion was lowest among non- drugs users (*N* = 35, 11%) compared to 22% among IDUs (*P* < 0.001). However, the IDUs had lower proportion of overweight/obese (*N* = 30, 8.1%) compared to 55% among the non- drug users. The proportion with low waist-for-hip ratio was highest among the IDUs (*N* = 274, 74%) while high waist-for-hip ratio was lowest in the same group of IDUs (*N* = 40, 11%), *P* < 0.001. One half had no anaemia (*N* = 379, 50%), 352 (47%) had mild/moderate anaemia while 21 (2.8%) had severe anaemia. The IDUs (54%) had higher proportion of mild/moderate anaemia compared to 37% non-IDUs and 40% non- drug users (*P* < 0.001) as shown in Table 3.

**Table 3:** Nutrition status using anthropometry stratified by drug use status of IDUs, non-

IDUs and non-DUs in Coastal Kenya between 2012–2013					
All participants		Drug Use Status			
Variables		Non- DUs	Non-IDUs	IDUs	*p-value*
	(N = 752)	(N = 306)	(N = 75)	(N = 371)	
Body Mass Index (BMI)					
< 18.5	263 (35)	69 (23)	25 (33)	169 (46)	< 0.001
18.5 to 24.9	420 (56)	180 (59)	40 (53)	200 (54)	
≥ 25	69 (9.2)	57 (19)	10 (13)	2 (0.5)	
MUAC*					
Underweight	130 (17)	35 (11)	15 (20)	80 (22)	< 0.001
Normal	391 (52)	103 (34)	27 (36)	261 (70)	
Overweight/obese	231 (31)	168 (55)	33 (44)	30 (8.1)	
Waist circumference^#^					
Normal	568 (76)	160 (52)	51 (68)	357 (76)	< 0.001
Overweight	184 (24)	146 (48)	24 (32)	14 (3.8)	
Waist-for-hip ratio^$^					
Low	467 (62)	143 (47)	50 (67)	274 (74)	< 0.001
Moderate	123 (16)	58 (19)	8 (11)	57 (15)	
High	162 (22)	105 (34)	17 (23)	40 (11)	
Anaemia^¶^					
None	379 (50)	174 (57)	43 (57)	162 (44)	0.001
Mild/moderate	352 (47)	122 (40)	28 (37)	202 (54)	
Severe	21 (2.8)	10 (3.3)	4 (5.3)	7 (1.9)	

All *p*-values are from Chi-square test; * Mid upper arm Circumference *Underweight MUAC < 23.9 cm, Normal is MUAC ≥ 24.0 to 28 cm for male and MUAC ≥ 24.0 to 28.3 cm for female, Overweight/obese is MUAC ≥ 28.1 cm for male and MUAC ≥ 28.4 cm for female; #Overweight waist circumference > 94 cm for male and > 80 cm for female; $low is defined as ratio < 0.8 for female & <0.95 for male, moderate is ratio 0.81 to 0.85 for female & 0.96 to 0.99 for male, high is ratio ≥ 0.86 for female and ≥ 1.0 for male; ¶No anaemia hemoglobin ≥ 12 g/dl for female or ≥ 13 g/dl for male, mild/moderate anaemia hemoglobin 8 to 11.9 g/dl for female and 8 to 12.9 g/dl for male, severe anaemia hemoglobin < 8.0 g/dl for both male & female

**Fig. 1:**
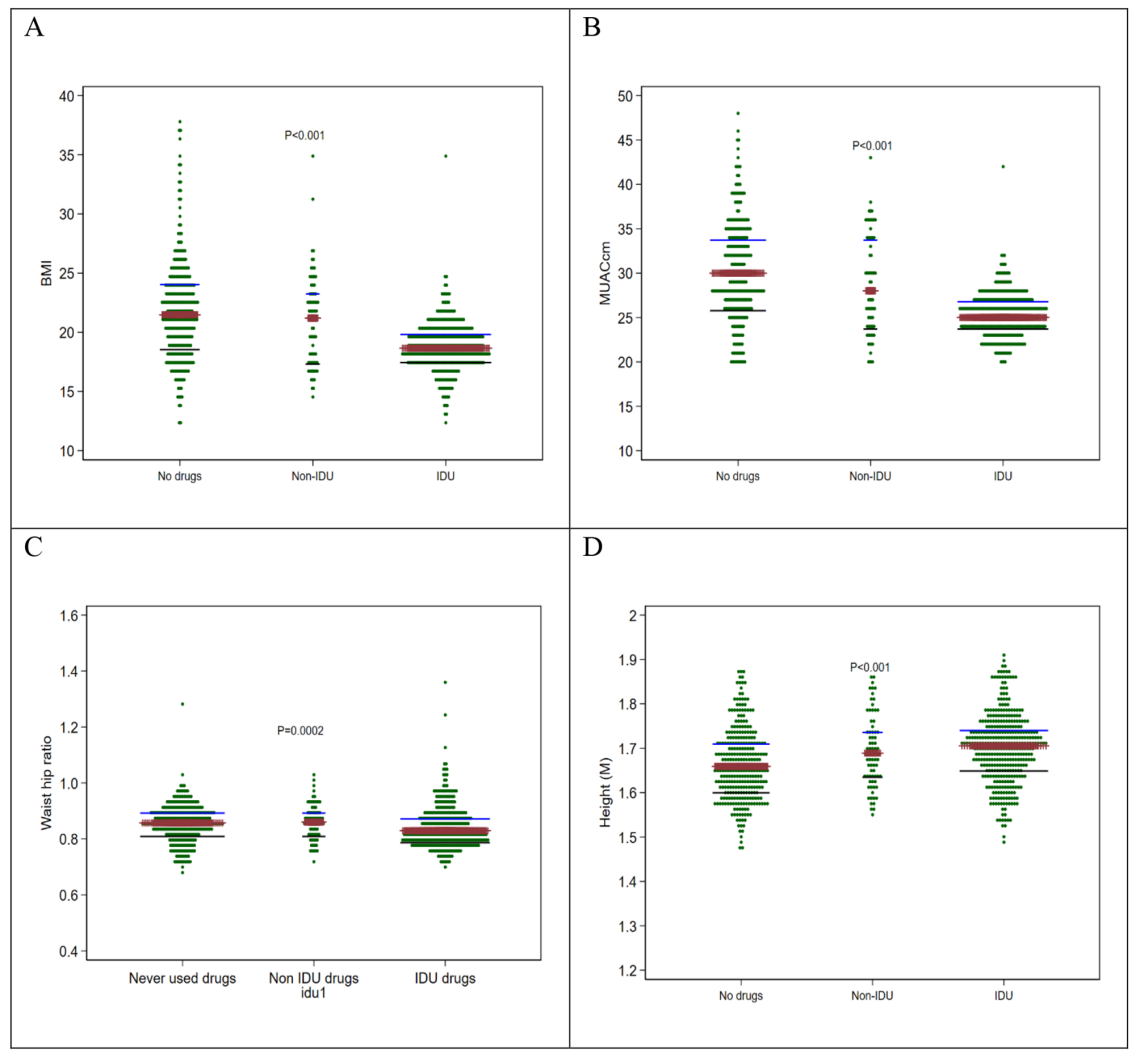
Dot plots of: (**a**) BMI, (**b**) MUAC (cm), (**c**) Waist-for-hip ratio and (**d**) Height (M) stratified by drug use status All the *p*-values are from Kruskal–Wallis test, the orange bar at the middle represent the median, the lower black line the 25% percentile and the upper blue line the 75% percentile.

All the continuous anthropometries shown in Supplementary Table 3a were significantly different across the three group (all Kruskal-Wallis test *p*-values < 0.05) (Fig. 1). However, in the pairwise comparison, height was not different between IDU drug users and the non-IDU users (adjusted *p*-value = 0.31), but all anthropometries were significantly different between IDU drug users and those never used drugs (Supplementary Table 3b). Similarly, weight, bust circumference, BMI and waist to hip ratio were not different between the never used drugs and the non-IDU users (Supplementary Table 3b).

The base models adjusted for sex and age are reported in Supplementary Table 4. In the multivariable models, IDUs (aRRR 2.83 (95%CI 1.84‒4.35)) and non-IDUs (aRRR 1.42 (95%CI 1.07‒1.88)) compared to non- drug users were positively associated with BMI < 18.5. However, being an IDU (aRRR 0.03 (95%CI 0.01‒0.07)) was negatively associated with BMI ≥ 25. Waist for hip ratio and height (per meter) were not associated with drug use status. Both IDUs and non-IDUs were positively associated with low MUAC while only IDUs were negatively associated with high MUAC. Being an IDU was positively associated with mild or moderate anaemia (aRRR 1.65 (95%CI 1.13‒2.41)). Conversely, non-IDUs were positively associated with severe anaemia (aRRR 1.69 (95%CI 1.16‒2.48)) as shown in Table 4.

**Table 4:** Multivariable analysis of association between drug use status and different anthropometric measurements in Coastal Kenya between 2012–2013

Body Mass Index (BMI)	BMI < 18.5 versus Normal BMI	*P*-value	BMI ≥ 25 versus Normal BMI	*p*-value
	Adjusted RRR (95% CI)		Adjusted RRR (95% CI)	
Non-drug users	Reference		Reference	
Non-IDUs	1.42 (1.07‒1.88)	**0.02**	0.79 (0.50‒1.24)	0.30
IDUs	2.83 (1.84‒4.35)	**< 0.001**	0.03 (0.01‒0.07)	**< 0.001**
**Waist-for-hip ratio**	**Moderate versus low**		**High versus low**	
	Adjusted RRR (95% CI)	*P*-value	Adjusted RRR (95% CI))	*p*-value
Non-drug users	Reference		Reference	
Non-IDUs	0.86 (0.70‒1.05)	0.15	1.22 (0.75‒2.01)	0.43
IDUs	0.77 (0.58‒1.02)	0.06	0.54 (0.15‒1.98)	0.35
**Mid upper arm circumference(cm)**	**Underweight versus normal**		**Overweight versus normal**	
	Adjusted RRR (95% CI)	*P*-value	Adjusted RRR (95% CI)	*p*-value
Non-drug users	Reference		Reference	
Non-IDUs	1.58 (1.33‒1.88)	**< 0.001**	0.78 (0.53‒1.15)	0.21
IDUs	1.39 (1.02‒1.88)	**0.04**	0.06 (0.03‒0.12)	**< 0.001**
**Anaemia levels**	**Mild/moderate versus None**		**Severe versus None**	
	Adjusted RRR (95% CI)	*P*-value	Adjusted RRR (95% CI)	*p*-value
Non-drug users	Reference		Reference	
Non-IDU users	0.70 (0.52‒0.95)	**0.02**	1.69 (1.16‒2.48)	**0.007**
IDU users	1.65 (1.13‒2.41)	**0.01**	0.90 (0.74‒1.09)	0.28
**Height in meters**				
			Adjusted Regression coefficient (95% CI)*	*p*-value
Non-drug users	¶		Reference	
Non-IDUs IDUs	¶ ¶		0.02 (-0.06 to 0.10) 0.02 (-0.01 to 0.04)	0.21 **0.06**

RRR; relative risk ratio, RRR adjusted for sex, age, education level, marital status, religion, monthly income, HIV status, sexual orientation, age of first sexual intercourse, history of sexual transmitted disease and recruiting hospital, RRR are from Multinomial logistic regression model, *Regression coefficient are from linear regression, ¶height was used as continuous dependent variable so there were group comparison (No results to report)

## Discussion

The study reports that IDUs in Coastal Kenya were more likely to be men with low level of education of Islamic faith in self-employment with a relatively higher income and currently single by the virtue of having never been married, separated, divorced or widowed in comparison to non-IDUs and non-drug users. These findings are mostly in agreement other studies carried out in the same region [29, 38, 39] with relatively higher income being an exception where most studies document the contrary [40]. The study reports that more IDUs tested HIV positive than the non-IDUs and non-drug users. Nevertheless the difference was in HIV status in relation to drug use status was not statistically significant. The relatively high proportion of HIV among IDUs could be attributed to high-risk injecting and sexual practices [29, 39, 41, 42]. Despite the free ARV programme in Kenya, fewer HIV positive IDUs were on ARVs with a lesser number having been on ARVs for more than three years as compared to their counter parts. Injection drug users are a hidden sub-population and consequently less likely to access healthcare or adhere to ARV regiment [28, 43]. Clinical signs were more present in the IDU group than their non-IDUs and non-drug users as more IDUs exhibited wheezy or congested chests. This could be partly due to effects of heroin on the respiratory system as opioids are respiratory depressants [44]. Wheezy and congested chests could also be due asthma which is aggravated by smoking tobacco products or marijuana and use of heroin or cocaine [45, 46].

In this study we found significant proportion of the participants were under-nourished with IDUs bearing the heaviest brunt. Being an IDU was directly associated with the low nutritional parameters like BMI, mid upper arm circumference (MUAC), waist for hip ratio and mild or moderate anaemia. However, IDUs were likely to be overweight based on waist circumference as a parameter. In the multivariable models being an IDU or a non-IDU predicted low BMI and low MUAC. On the other hand, injection drug use was positively associated with mild or moderate anaemia while non-injection drug use was positively associated with severe anaemia.

Findings on low BMI in this study are supported by existing data that report low BMI among injecting drug users [11, 47]. Low BMI is an indicator of leanness and the contrary implies whole body adiposity. Low BMI can be attributed to suppressed appetite due to the use of heroin, khat and tobacco products as reported in a similar sub-populations elsewhere [15–17, 21, 48, 49]. Low BMI could also be due to vomiting and diarrhoea as reported in the study possibly due to withdrawal symptoms of heroin [50] or underlying illnesses like HIV/AIDS [4]. Additionally, food insecurity arising from competing needs between food and drugs could be a possible contributing factor to low BMI among IDUs. In Chennai, Vancouver and Los Angeles as well as San Francisco (both in United States of America) studies among IDUs reported food insecurity [4, 8, 51].

Larger waist circumference is a nutritional marker for central adiposity. In this study, larger waist circumference could be due the fact that people addicted to opiates tend to experience increased preferences for sweet tasting food. Consequently, their diet is inclined towards refined carbohydrates and fats, meals high in sugar as well as alcohol thus consuming more calories that are low in essential nutrients [12, 49, 52, 53]. Alternatively, a larger waist circumference can be attributed to metabolic effects of nicotine on body weight or composition like low adiponectin levels which modulates insulin sensitivity [48]. Reduced adiponectin levels are one of the known causes of insulin resistance which alters body composition including increased visceral fat [54]. In one of our previous studies we demonstrated that HIV mono-infected IDUs had suppressed adiponectin levels [27].

Even though more IDUs and non-IDUs had a relatively larger waist circumference than the non-drug users, their waist for hip ratio was lower. This could be due to active tobacco smoking as reported by some of the participants and second hand smoking by virtue of association. In general, smokers tend to be leaner than non-smokers [48] possibly due to the effects of nicotine which is believed to increase metabolic rate and therefore increase in energy expenditure [55]. Elsewhere tobacco smoking has been linked to central adiposity and not overall obesity as smokers tend to have a low BMI [56, 57]. Additionally, during active heroin use most users tend to eat less frequently due to reduced appetite thus the tendency to be lean [49]. Contrary to these findings a study in nineteen Norwegian Counties reported a lower hip circumference but a higher waist circumference and waist-hip-ratio in current smokers [58].

In the present study, low MUAC which is a nutritional marker for wasting or acute malnutrition was documented among IDUs and non- IDUS as opposed to non-drug users. This could be due to food insufficiency or insecurity which is common in this sub-population. This has been reported in among PWIDs in Canada, in West Virginia (United States of America) as well as in Chennai [4, 5, 8, 59]. Hunger among drug users can be attributed to their low socio-economic status, chaotic lifestyle, withdrawal symptoms, mouth sores and competing demands for food and recreational drugs [5, 8, 60].

In this study, slightly more than a half of the IDUs exhibited mild or moderate anaemia. These findings are supported by our previous work which reported severe anemia and microcytic hypochromic anaemia among HIV-1 and Mycobacterium tuberculosis co-infected IDUs [30, 31]. Anaemia in this sub-population could be due to consumption of foods of low nutritive value. In Iran, a study documented that IDUs exhibited preference for ready to eat snacks and were unlikely to consume fruits, vegetables, dairy products and meat [11]. In Brazil, a study among IDUs reported low hemoglobin and hematocrit levels which are associated with protein-energy malnutrition as well as anaemia [61]. Poor eating habits can also be due to being single [9] thus no motivation to prepare food or unstable housing consequently lack of kitchen facilities for proper food preparation as well as storage. Overall, the implications of these results are that incorporation of iron supplements in the nutritional management schedules IDUs would reduce the risk of anaemia.

The strength of this study is that it was the first of its kind along the Coastal Kenya and provides data about nutritional status of IDUs compared to non-IDUS which is important in developing appropriate interventions. Limitations of the study include limited generalizability because drug use was self-reported. This could have prejudiced the findings through introduction of social desirability bias. The study design does not provide evidence for temporal relationship because data on drug use and nutritional status were collected simultaneously and once. Additionally it is not possible to tell if under nutrition was heightened by poverty or cultural dietary orientation.

## Conclusion

Findings from this study indicate that injection drug use heightened the risk of under-nutrition as evidenced by low BMI, MUAC, waist for hip ratio and anaemia. However, PWIDs were more likely to be overweight based on waist circumference as a parameter. People who inject drugs have a higher risk for under nutrition and should be targeted with appropriate interventions.

### Electronic supplementary material

Below is the link to the electronic supplementary material.

Supplementary Material 1

Supplementary Material 2

Supplementary Material 3

## Data Availability

All data generated or analysed during this study are included in this published article (Supplementary dataset. Nutritional status of PWIDs in Coastal Kenya.csv).

## Abbreviations

AIDS: Acquired Immunodeficiency Syndrome
ARRR: Adjusted Relative Risk Reduction
BMI: Body Mass Index
DSM-5-TR: Diagnostic and Statistical Manual of Mental Disorders
HIV: Human immunodeficiency virus
IDU: Injection Drug Use (r)
IQR: Interquartile Range
KES: Kenya Shillings
MOH: Ministry of Health
MUAC: Mid-Upper Arm Circumference
NACADA: National Authority for the Campaign against Drug Abuse
NASCOP: National AIDS & STI Control Program
Non- IDU: Non-Injection Drug Use (r)
Non-DU: Non- Drug Use (r)
PWID: People Who Inject Drugs
PWUD: People Who Use Drugs
UNODC: United Nations Office on Drugs and Crime
WHO: World Health Organization
WHR: Waist-To-Hip Ratio
