# Supplementary material for: Nutritional status of people who inject drugs in Coastal Kenya: a cross-sectional study
Source: BMC Nutr. 2024 Apr 4;10:55. doi: 10.1186/s40795-024-00851-z (PMC10996164; doi:10.1186/s40795-024-00851-z)
Supplement: Supplementary file 1 — Supplementary Material 1 [file 40795_2024_851_MOESM1_ESM.docx]

# **Supplementary Materials**

## Supplementary Table 1: Summary of self-reported injecting and non-injecting drugs used by IDUs in coastal Kenya.

| **Injecting Drugs** | **IDUs (N=371)** | **%** | | |
| --- | --- | --- | --- | --- |
| Heroin | 303 | 82 | |  |
| Cocaine | 61 | 16 | |  |
| Heroin and cocaine | 7 | 1.9 | |  |
| Others^#^ | 40 | 11 | |  |
| Non injecting drugs |  |  | |  |
| Alcohol | 119 | 32 | |  |
| Khat | 112 | 30 | |  |
| Tobacco | 254 | 68 | |  |
| Rohypnol | 211 | 57 | |  |
| Brown sugar* | 49 | 13 | |  |
| Marijuana | 183 | 49 | |  |
| Cocktail^$^ | 118 |  | 32 |  |

All results are N (%), # Others include injecting diazepam (N=26) and Rohypnol (N=14), *Non-injectable heroin, ^$^ Mixture of cigarette and marijuana, ^¶^The numbers are more than 371 because some were taking more than one drug.

## Supplementary Table 2: Summary of self-reported drugs consumed by non-injecting drug users in coastal Kenya.

| **Non-injecting Drugs^¶^** | N (N=75) |  | % |
| --- | --- | --- | --- |
| Alcohol | 39 |  | 52 |
| Khat | 25 |  | 33 |
| Tobacco | 25 |  | 33 |
| Rohypnol | 3 |  | 4 |
| Brown sugar* | 3 |  | 4 |
| Marijuana | 5 |  | 6.7 |
| Cocktail^$^ | 1 |  | 1.3 |

All results are N (%). *Non-injectable heroin, ^$^ Mixture of cigarette and marijuana, ^¶^The numbers are more than 75 because some were taking more than one drug.

## Supplementary Table 3a: Continuous anthropometry stratified by drug use status in Coastal Kenya

| Anthropometry, median (IQR) |  | **Drug use status** | | | |
| --- | --- | --- | --- | --- | --- |
|  | **All participants (N=752)** | **Never used drugs (N=306)** | **Non-IDU drugs (N=75)** | **IDU drugs (N=371)** | **P-value** |
| Weight (Kg) | 56 (51‒63) | 60 (54‒67) | 60 (51‒66) | 54 (50‒58) | <0.001 |
| Height (M) | 1.69 (1.63‒1.74) | 1.66 (1.61‒1.72) | 1.69 (1.64‒1.74) | 1.71 (1.65‒1.75) | <0.001 |
| Waist circumference (Cm) | 78 (72‒86) | 84 (75‒91) | 80 (71‒890) | 75 (71‒79) | <0.001 |
| Hip circumference (Cm) | 93 (87‒100) | 99 (90‒106) | 96 (85‒105) | 90 (87‒94) | <0.001 |
| Mid upper arm circumference (Cm) | 27 (24‒30) | 30 (26‒34) | 28 (24‒34) | 25 (24‒27) | <0.001 |
| Bust circumference (Cm) | 87 (82‒94) | 92 (84‒98) | 92 (79‒99) | 85 (81‒88) | <0.001 |
| Body Mass Index (BMI) | 19.7 (17.8‒22.1) | 21.6 (18.9‒24.3) | 21.2 (17.4‒23.4) | 18.7 (17.6‒29.0) | <0.001 |
| Waist to hip ratio | 0.85 (0.80‒0.90) | 0.86 (0.82‒0.90) | 0.86 (0.82‒0.90) | 0.83 (0.80‒0.88) | 0.0002 |

All p-values are from Kruskal–Wallis test, all the results are median (IQR), IQR; Interquartile range.

## Supplementary Table 3b: Post-hoc correction for multiple Continuous anthropometry comparisons.

| Anthropometry | **Corrected P-values** | | |
| --- | --- | --- | --- |
|  | **IDU drugs versus Never used drugs** | **IDU drugs versus Non-IDU drugs** | **Never used drugs versus Non-IDU drugs** |
| Weight (Kg) | <0.001 | <0.001 | 0.87 |
| Height (M) | <0.001 | 0.31 | 0.011 |
| Waist circumference (Cm) | <0.001 | 0.0003 | 0.024 |
| Hip circumference (Cm) | <0.001 | 0.0003 | 0.013 |
| Mid upper arm circumference (Cm) | <0.001 | <0.001 | 0.012 |
| Bust circumference (Cm) | <0.001 | 0.0002 | 0.104 |
| Body Mass Index (BMI) | <0.001 | <0.001 | 0.070 |
| Waist to hip ratio | 0.0005 | 0.02 | 0.72 |

The corrected P-values were from post hoc adjustment for each pairwise comparison conducted using the `holm’ method implemented by *FSA* package in R.

## Supplementary Table 4: Effects of drug use status on different anthropometric measurement (Base model) in Coastal Kenya

| **Body Mass Index (BMI)** |  | |  | |
| --- | --- | --- | --- | --- |
|  | BMI <18.5 versus Normal BMI | | BMI≥25 versus Normal BMI | |
|  | Adjusted RRR (95%CI) | P-value | Adjusted RRR (95%CI) | P-value |
| Never used drugs | Reference |  | Reference |  |
| Non-IDU users | 1.47 (1.14‒1.88) | 0.003 | 0.80 (0.46‒1.39) | 0.44 |
| IDU users | 2.03 (1.65‒2.51) | <0.001 | 0.04 (0.03‒0.07) | <0.001 |
| **Waist-for-hip ratio** |  |  |  |  |
|  | Moderate versus low | | High versus low | |
|  | Adjusted RRR (95%CI) | P-value | Adjusted RRR (95%CI) | P-value |
| Never used drugs | Reference |  | Reference |  |
| Non-IDU users | 0.84 (0.81‒0.87) | <0.001 | 1.09 (0.90‒1.32) | 0.38 |
| IDU users | 1.24 (1.08‒1.39) | 0.001 | 0.48 (0.27‒0.85) | 0.01 |
| **Mid upper arm circumference (cm)** | | | | |
|  | Underweight versus normal | | Overweight versus normal | |
|  | Adjusted RRR (95%CI) | P-value | Adjusted RRR (95%CI) | P-value |
| Never used drugs | Reference |  | Reference |  |
| Non-IDU users | 1.78 (1.25‒2.53) | 0.001 | 0.79 (0.52‒1.21) | 0.28 |
| IDU users | 1.02 (0.59‒1.79) | 0.94 | 0.07 (0.06‒0.08) | <0.001 |
| **Anemia levels** |  |  |  |  |
|  | Mild/moderate versus None | | Severe versus None | |
|  | Adjusted RRR (95%CI) | P-value | Adjusted RRR (95%CI) | P-value |
| Never used drugs | Reference |  | Reference |  |
| Non-IDU users | 0.87 (0.73‒1.06) | 0.16 | 2.11 (1.06‒4.20) | 0.03 |
| IDU users | 1.62 (1.10‒2.40) | 0.02 | 0.94 (0.23‒3.78) | 0.93 |
| **Height in meters** |  |  |  |  |
|  |  |  | Adjusted Regression (95% CI) | P-value |
| Never used drugs | ¶ |  | Reference |  |
| Non-IDU users | ¶ |  | 0.02 (-0.04 to 0.07) | 0.16 |
| IDU users | ¶ |  | 0.02 (-0.02 to 0.07) | 0.11 |

RRR; relative risk ratio, RRR adjusted for sex, age and recruiting hospital, RRR are from Multinomial logistic regression model.
