# Supplementary material for: Nutritional status of people who inject drugs in Coastal Kenya: a cross-sectional study
Source: BMC Nutr. 2024 Apr 4;10:55. doi: 10.1186/s40795-024-00851-z (PMC10996164; doi:10.1186/s40795-024-00851-z)
Supplement: Supplementary file 2 — Supplementary Material 2 [file 40795_2024_851_MOESM2_ESM.docx]

# CUT-OFF POINTS FOR WHR, WC, MUAC AND ANAEMIA

## MUAC

| **Gender** | **MUAC (cm)** | **Classification** |  |
| --- | --- | --- | --- |
| **Male** | > or=23 | Normal |  |
|  | <23 | Malnourished |  |
| **Female** | > or =22 | Normal |  |
|  | <22 | Malnourished |  |

Tang, Alice M.; Dong, Kimberly; Deitchler, Megan; Chung, Mei; Maalouf-Manasseh, Zeina; Alison Tumilowicz, Alison; Wanke, Christine. 2013. Use of Cutoffs for Mid-Upper Arm Circumference (MUAC) as an Indicator or Predictor of Nutritional and HealthRelated Outcomes in Adolescents and Adults: A Systematic Review. Washington, DC: FHI 360/FANTA.

<https://www.fantaproject.org/sites/default/files/resources/MUAC%20Systematic%20Review%20_Nov%2019.pdf>

## WAIST CIRCUMFERENCE

| Indicator | Cut-off points Risk of metabolic complications |
| --- | --- |
| Waist circumference | >94 cm (M); >80 cm (W) |
| Waist circumference | >102 cm (M); >88 cm (W) |
| Waist–hip ratio | ≥0.90 cm (M); ≥0.85 cm (W) |

M, men; W, women

Source: WHO, 2011. Waist circumference and waist-hip ratio: report of a WHO expert consultation. <https://www.who.int/publications/i/item/9789241501491>

## BMI


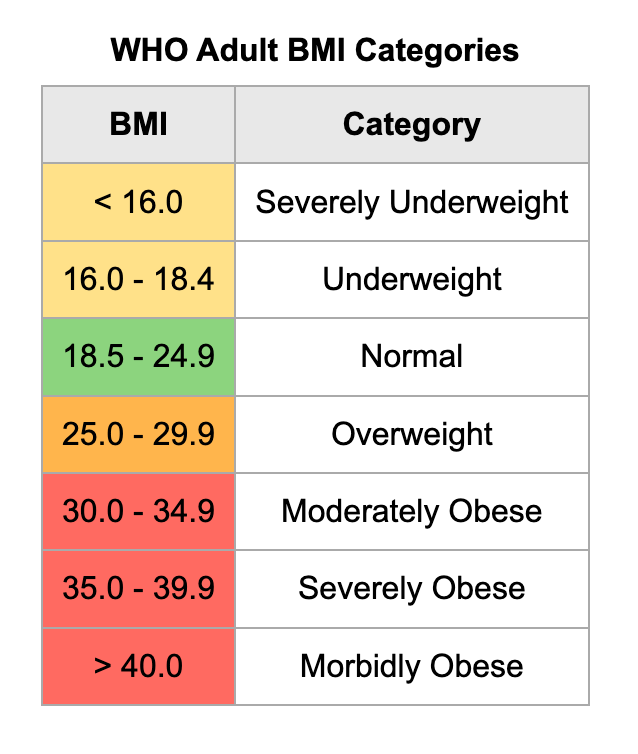


WHO, 2023. <https://www.who.int/europe/news-room/fact-sheets/item/a-healthy-lifestyle---who-recommendations>

## HAEMOGLOBIN

| **Population** | **Non anemia** | **Mild** | **Moderate** | **Severe** |
| --- | --- | --- | --- | --- |
| Children 6 - 59 months of age | 110 or higher | 100-109 | 70-99 | lower than 70 |
| Children 5 - 11 years of age | 115 or higher | 110-114 | 80-109 | lower than 80 |
| Children 12 - 14 years of age | 120 or higher | 110-119 | 80-109 | lower than 80 |
| Non-pregnant women  (15 years of age and above) | 120 or higher | 110-119 | 80-109 | lower than 80 |
| Pregnant women | 110 or higher | 100-109 | 70-99 | lower than 70 |
| Men (15 years of age and above) | 130 or higher | 110-129 | 80-109 | lower than 80 |

WHO, 2011. Haemoglobin concentrations for the diagnosis of anaemia and assessment of severity. Vitamin and Mineral Nutrition Information System. Geneva, World Health (<http://www.who.int/vmnis/indicators/haemoglobin>).
